# Supplementary material for: Chromatin accessibility landscape of pediatric T‐lymphoblastic leukemia and human T‐cell precursors
Source: EMBO Mol Med. 2020 Aug 5;12(9):e12104. doi: 10.15252/emmm.202012104 (PMC7507092; doi:10.15252/emmm.202012104)
Supplement: Supplementary file 1 — Appendix [file EMMM-12-e12104-s001.pdf]

APPENDIX

Table of Contents

Appendix Figure S1

Number of identified TF binding motifs in seven maturation stages of human T-cell precursors (DN2 (n=5), DN3 (n=5), ISP (n=5), DPCD3- (n=6), DPCD3+ (n=6), SPCD4+ (n=5), SPCD8+ (n=5)) **(A)** and in T-ALLs (n=19) **(B)**. On the box plots horizontal lines indicate median, lower and upper limits of each box correspond to the first and third quartiles (the 25th and 75th percentiles) and the lower and upper whiskers extend from min to max.

Appendix Figure S2

Fraction of each maturation stage in thymus as measured by FACS. Mean fraction of the populations isolated from six thymi are used for plotting

Appendix Figure S3

Leave-one-out cross-validation of signature matrices. Heatmaps represent the fraction of each maturation stage in the sorted populations isolated from six thymi as predicted by CIBERSORT. T(n) stands for the thymus donor. In **(A)** predictions were performed using 2,021 signature OCRs collected from seven sorted populations of human T-cell precursors and in **(B)** using 2,823 signature peaks collected from combined consecutive stages.

Appendix Figure S4

Internalization of CD3 surface marker. FACS plots show the percentage of CD3+ cells in the sorted DPCD3+ population. CD3 on the x-axis, viability dye 7AAD on the y-axis. The purity of sorted DPCD3+ population was checked after 15 minutes **(left)** and after the usual 8 hours of sorting **(right)** period.

Appendix Figure S5

Number of signature peaks assigned to each stage of maturation of thymic T-cell precursors. For equal representation of maturation stages, functional enrichment analysis was performed in three groups that were condensed from five consecutive groups

Appendix Figure S6

Number of stage-specific and shared TFs. **(A)** TFs whose binding motifs are enriched in at least one maturation stage of thymic T-cell precursors. (ln(odds ratio) >0 and FDR <0.05). **(B)** TFs whose binding motifs are depleted in at least one developmental stage (ln(odds ratio) <0 and FDR <0.05).

Appendix Figure S7

Principal component 1 contributions (y-axis) of the top 34 peaks to the separation of healthy T-cell precursors and T-ALLs. Both TSS and distal peaks are considered while quantifying contributions. Peaks are annotated by nearest genes (x-axis).

Appendix Figure S8

Microarray expression profiling of sorted human thymocytes. Differential expression of *DAB1*, *HBA1* (negative control), and *GAPDH* (positive control).

Appendix Figure S9

Intensity of DAB1 expression in publicly available datasets generated with Affymetrix microarray (U133). Raw intensity values (y-axis) are log2-transformed. Cell types (x-axis) are indicated by color. Orange: brain cells where high DAB1 expression is expected. Gray: normal blood cells, lymphocytes or B-cells. Green: ALL cells.

Appendix Figure S10

Unsupervised hierarchical clustering based on the VST normalized RNA read counts (each row is also normalized by the row mean) of 292 gene-peak pairs in 19 T-ALLs. Orange triangles indicate three patients with a dominant DN2 profile, high SPI1 expression and motif counts but no DAB1 expression.

Appendix Figure S11

Spearman's rank correlation (R=0.71) of normalized RNA read counts (x-axis) and predicted SPI1 binding motif counts (y-axis). Blue indicates three patients with a dominant DN2 profile, high SPI1 expression and motif counts but no DAB1 expression. Red indicates remaining 16 patients

Appendix Figure S12

FACS gating strategy. Thymocytes were stained for CD7, CD1a, CD34, CD38, CD3, CD4 and CD8 surface markers. After gating singlets, viable CD7 positive T-cells were gated to prevent any contamination with stromal cells such as epithelial cells from the cortex and medulla or dendritic cells. In the CD34 positive fraction, two populations were identified based on CD1a expression: CD1a negative (DN2) and CD1a positive (DN3). Out of the CD34 negative cells, CD3 negative and CD3 positive cells were gated. From the CD3 negative fraction two populations were identified: CD4 positive cells (ISP/DN4) and CD4 positive CD8 positive (DPCD3-). In turn, 3 populations were identified from the CD3 positive fraction: CD4 positive CD8 positive (DPCD3+), CD4 positive (SPCD4+) and CD8 positive (SPCD8+).

Appendix table S1

Fraction of sorted populations in six thymus donors.

Appendix Figure S1

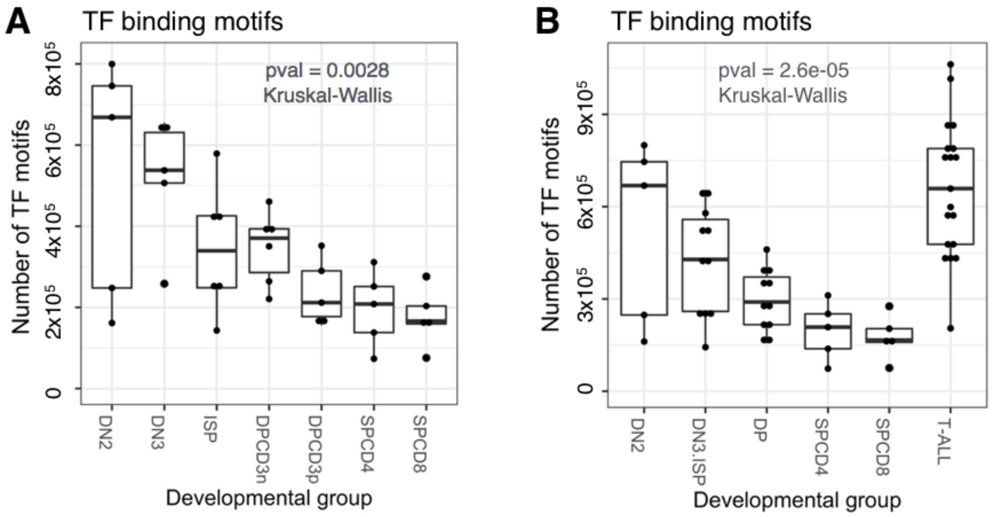

Appendix Figure S2

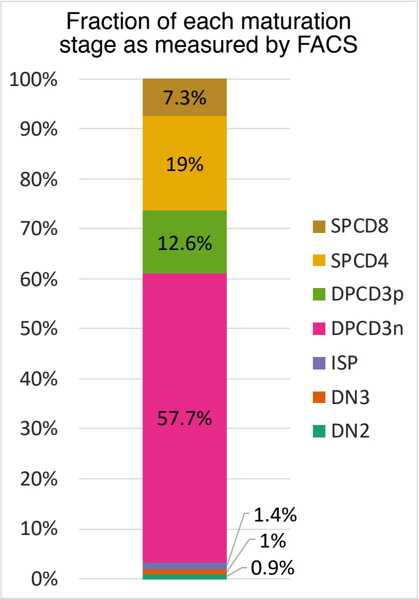

Appendix Figure S3

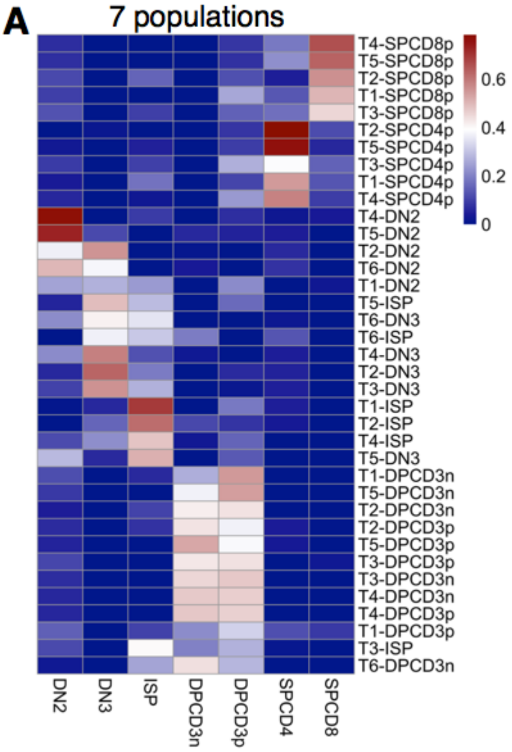

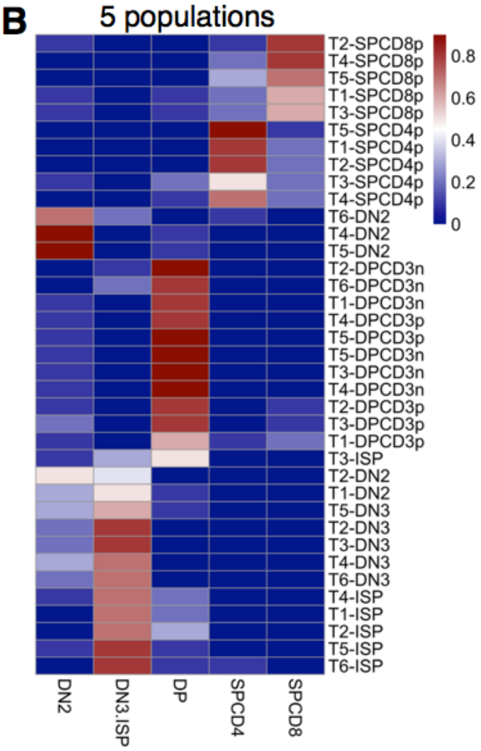

Appendix Figure S4

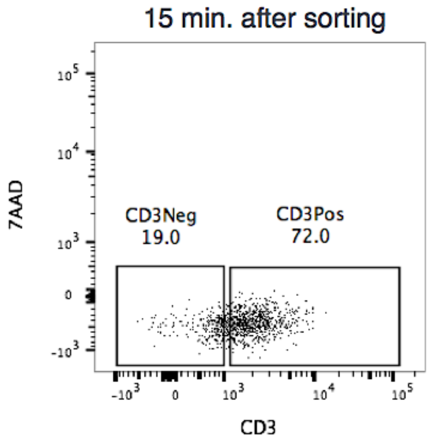

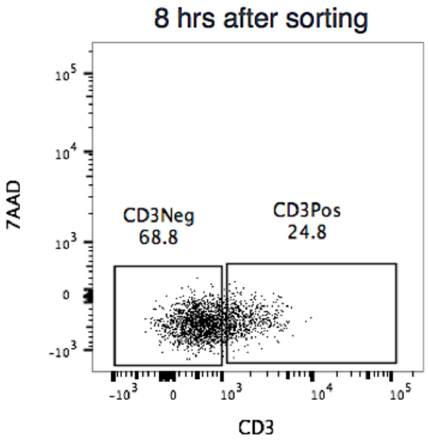

Appendix Figure S5

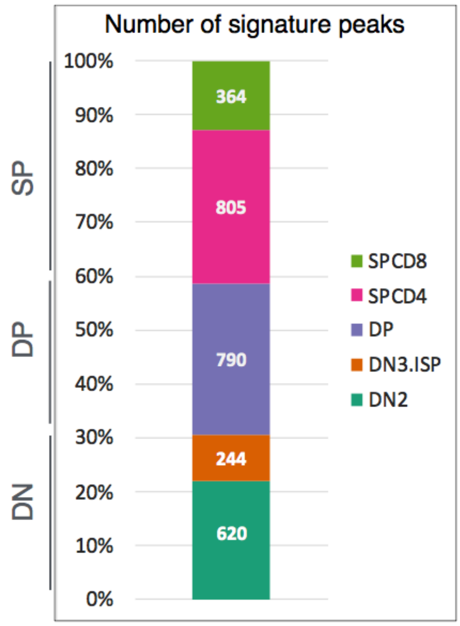

Appendix Figure S6

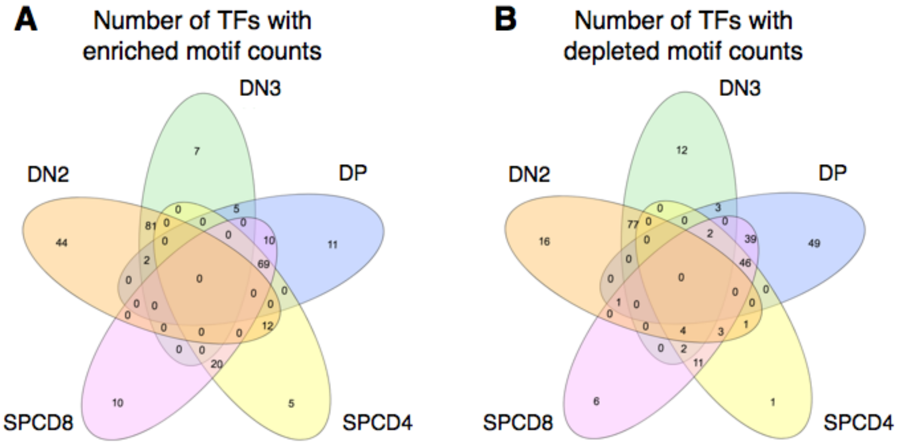

Appendix Figure S7

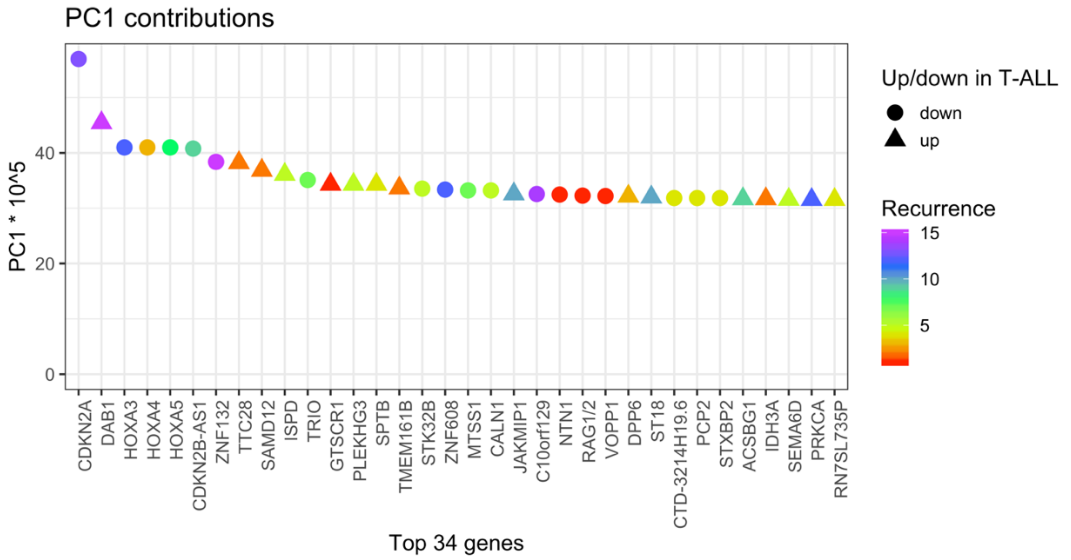

Appendix Figure S8

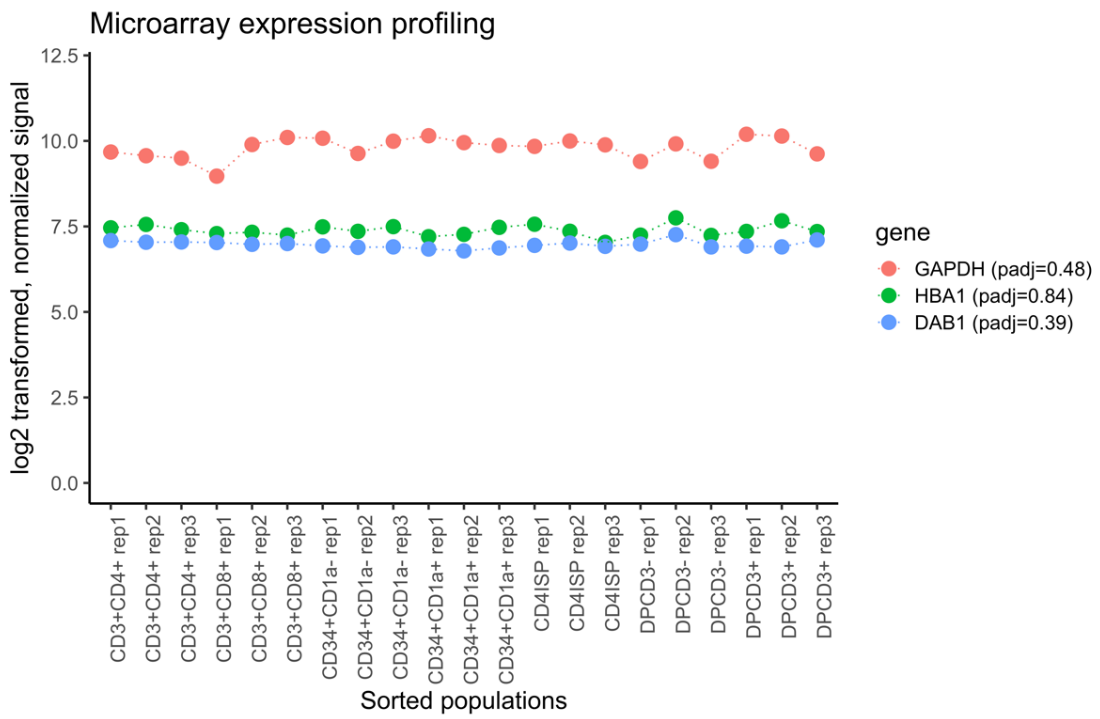

Appendix Figure S9

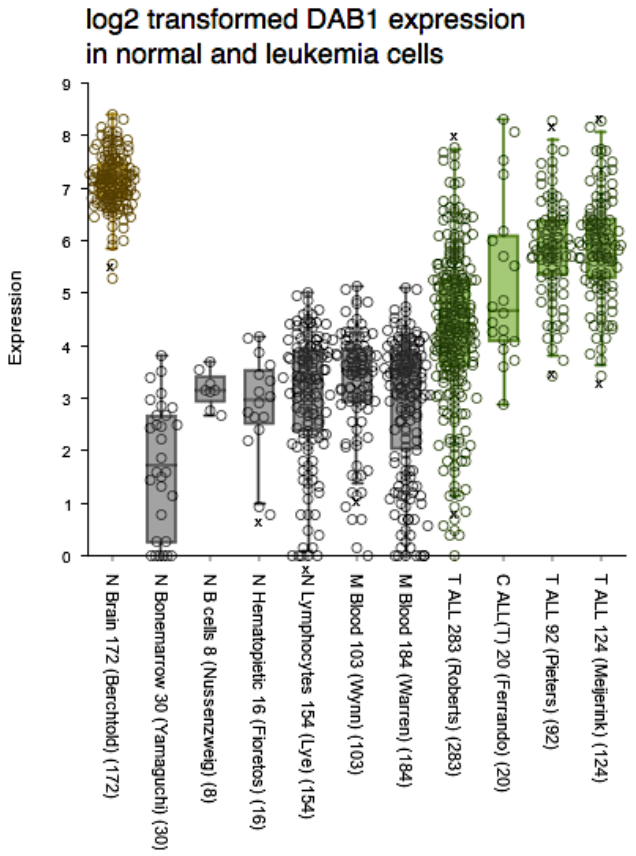

Appendix Figure S10

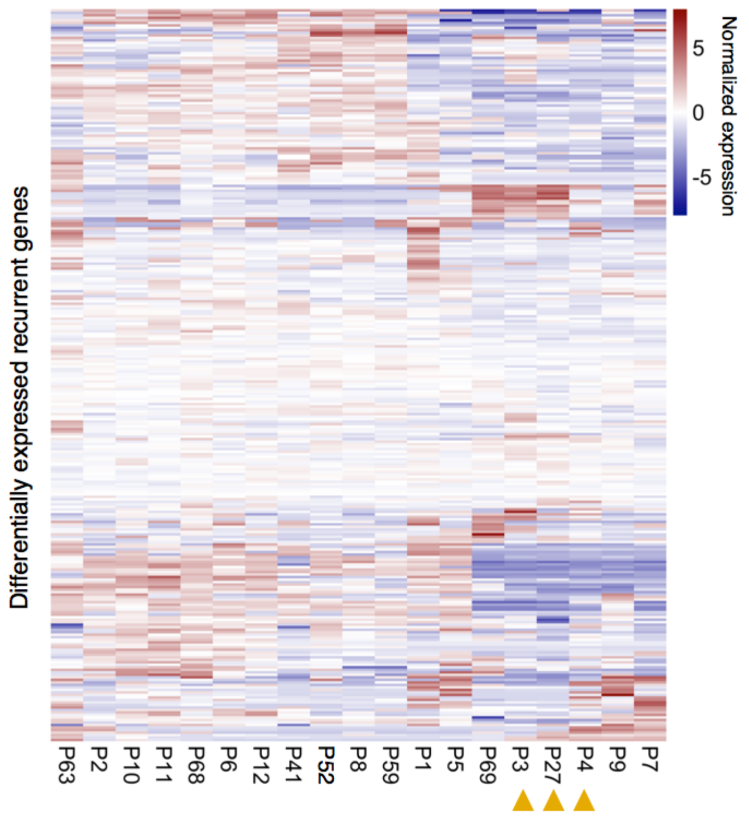

Appendix Figure S11

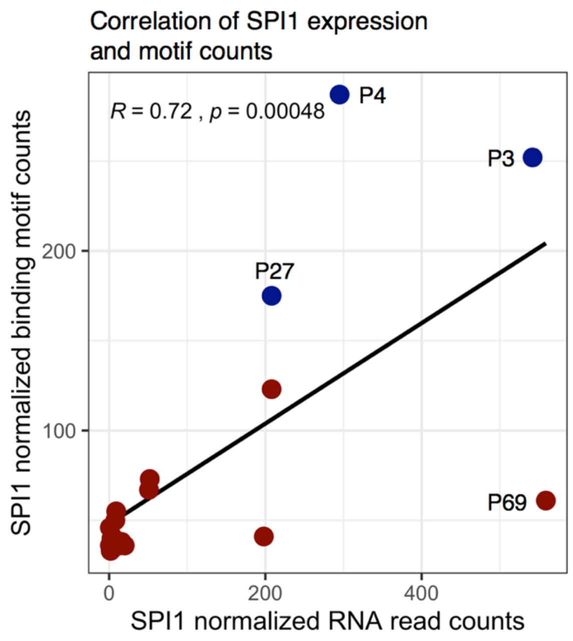

Appendix Figure S12

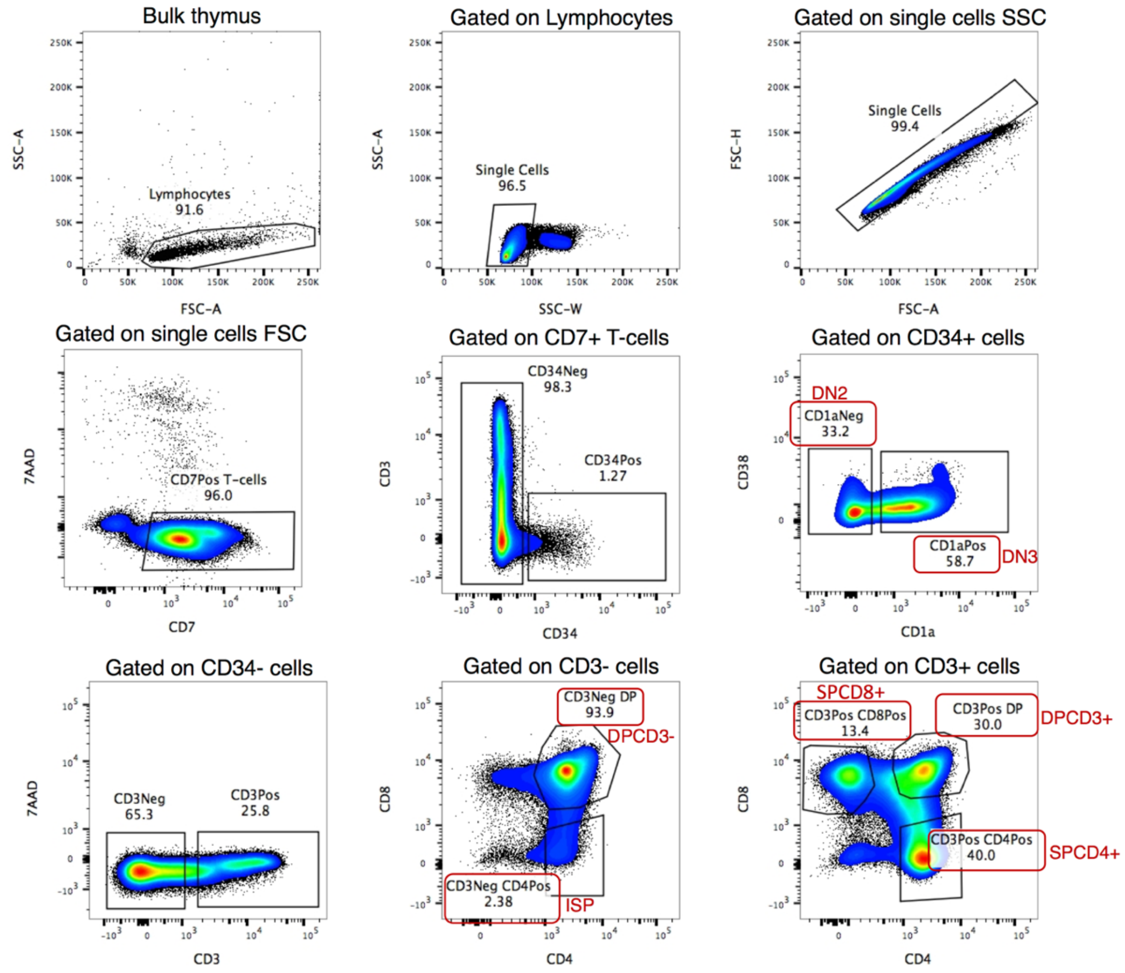

Appendix Table S1

Donor	DN2 (%)	DN3 (%)	ISP (%)	DPCD3N (%)	DPCD3P (%)	CD4 (%)	CD8 (%)	Sex	Age
**T1**	1.3	0.2	0.8	73.8	7.6	12.1	4.0	M	0.3
**T2**	0.9	1.2	1.3	64.4	12.2	11.5	8.4	F	1.3
**T3**	1.5	1.2	2.3	54.6	19.6	15.1	5.7	M	11.6
**T4**	0.7	1.9	1.8	52.2	22.9	14.1	6.5	F	6.2
**T5**	0.8	1.0	1.7	67.5	10.6	14.0	4.4	F	1.6
**T6**	0.3	0.5	0.8	33.4	2.7	47.4	15.0	M	0.0
**Mean**	**0.9**	**1.0**	**1.4**	**57.7**	**12.6**	**19.0**	**7.3**		**3.5**

Legend

Excluded from analysis because quality controls after sequencing identified contamination by other populations.

Not sequenced because quality controls before sequencing identified poor library quality.
