# Supplementary material for: Chromatin accessibility landscape of pediatric T‐lymphoblastic leukemia and human T‐cell precursors
Source: EMBO Mol Med. 2020 Aug 5;12(9):e12104. doi: 10.15252/emmm.202012104 (PMC7507092; doi:10.15252/emmm.202012104)
Supplement: Supplementary file 2 — Expanded View Figures PDF [file EMMM-12-e12104-s002.pdf]

Expanded View Figures

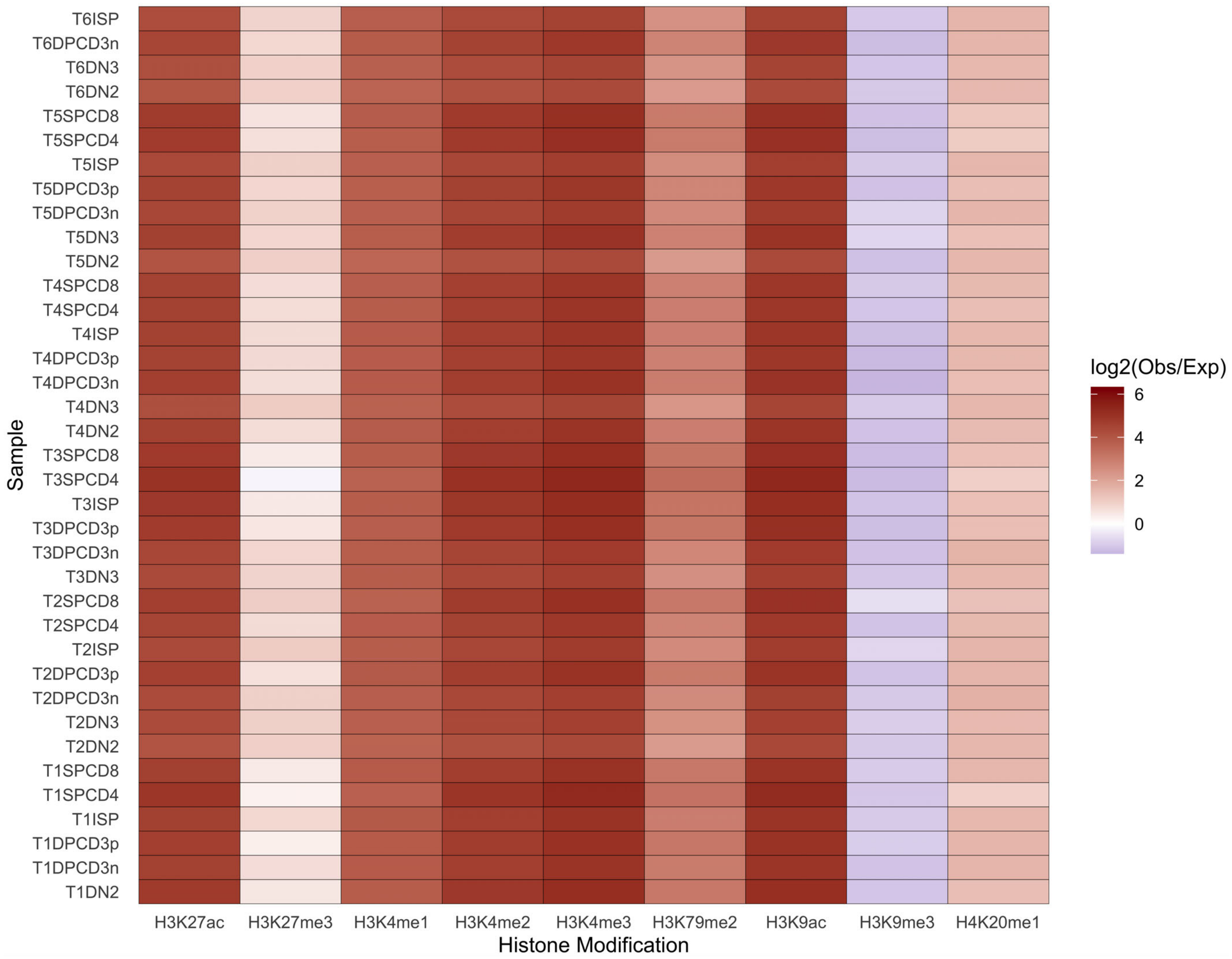

Figure EV1. Comparison of the ATAC-Seq peaks with the chromatin immuno-precipitation DNA-sequencing.
Heatmap showing degree of overlap between the ATAC-Seq peaks and the active promoters and enhancers detected in histone methylation/acetylation analysis by chromatin immunoprecipitation and sequencing of the T-cell leukemia cell line DND-41. Expected values were computed based on the randomly shuffled peaks.

© 2020 The Authors

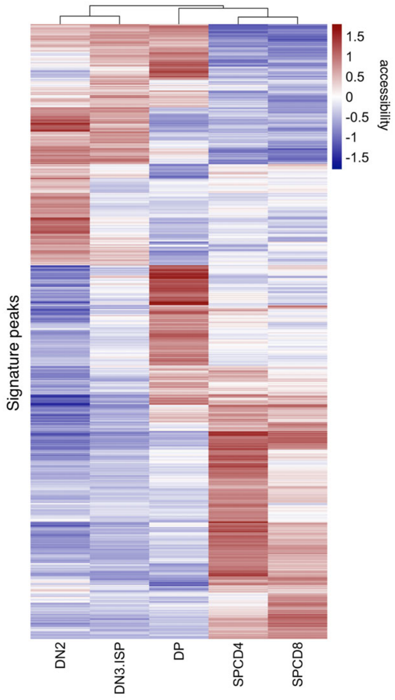

Figure EV2. Unsupervised hierarchical clustering based on the VST normalized read counts (each row is also normalized by the row mean) of the 2,823 signature OCRs.

Five groups represent the developmental stages which were formed after merging ATAC-Seq data of the populations that showed most commonalities, namely DN3 & ISP and DPCD3^{-} & DPCD3^{+}.

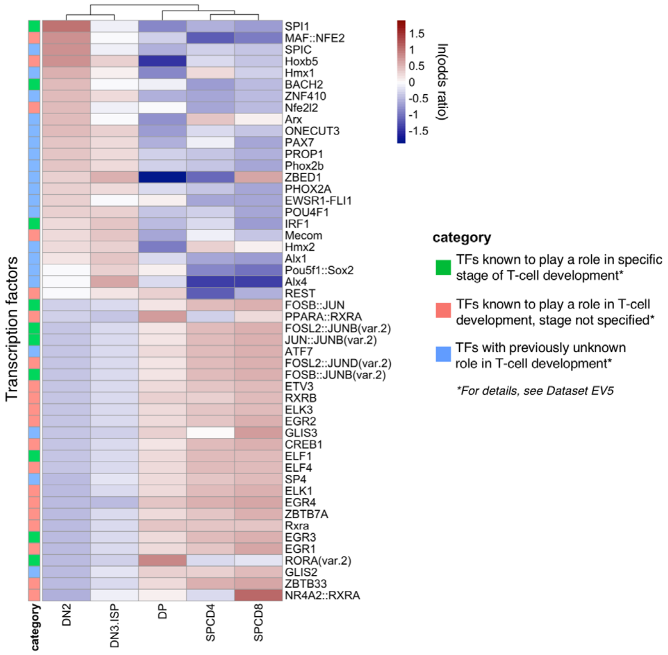

Figure EV3. Global TF motif enrichment analysis performed on the ATAC-Seq peaks alone.

Heatmap of ln(odds ratio) of the 50 TFs showing the most differential abundance of binding motifs in open chromatin regions. TFs with an SD of the motif counts < 10 are excluded.

© 2020 The Authors

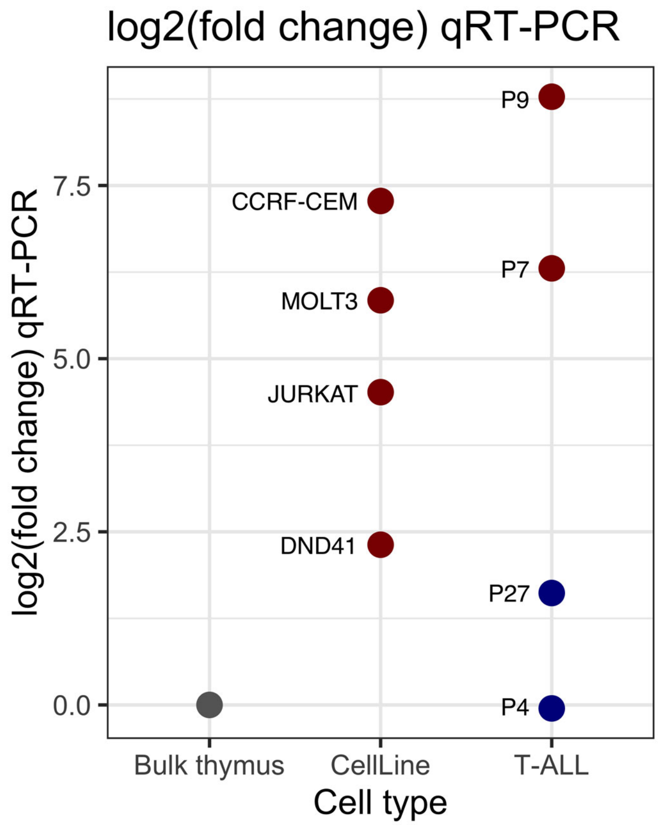

Figure EV4. Differential *DAB1* expression in T-ALL cell lines and in four primary T-ALLs in comparison to thymus.

Red indicates up and blue indicates downregulation of *DAB1* in comparison to bulk thymus (gray). qPCR reactions were run in triplicates and mean $\log_2$ fold changes are shown on the plot. RPL19 gene was used as a housekeeping control for the C_T normalization.

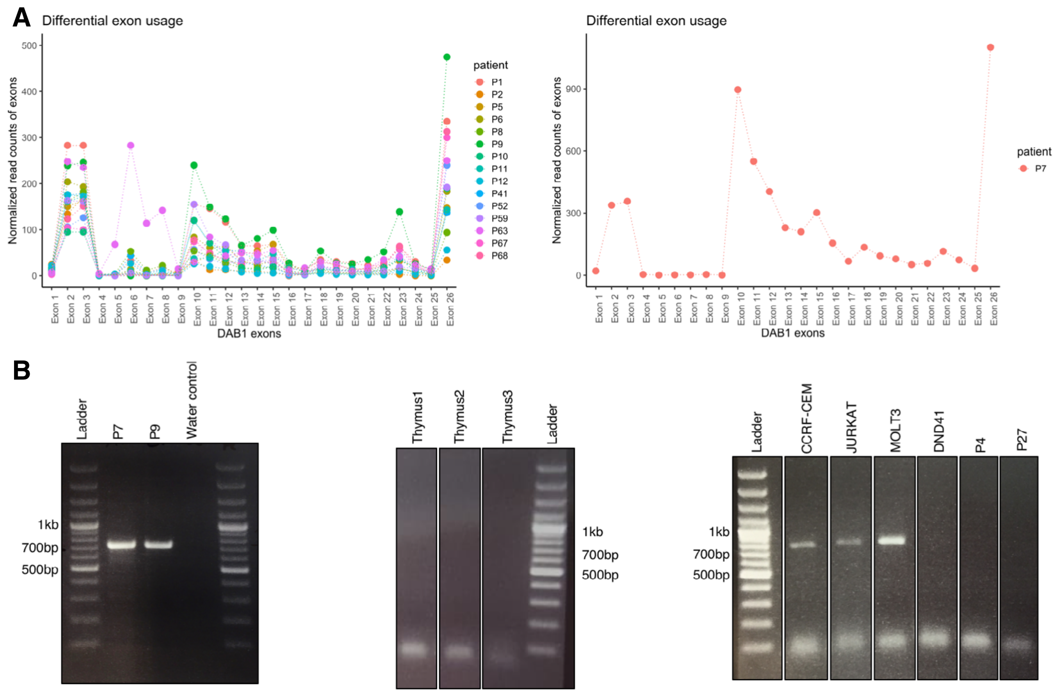

Figure EV5.

◀ **Figure EV5. Differential exon usage analysis of *DAB1*.**

A Expression of *DAB1* exons (*x*-axis) in *DAB1*(+) patients. Read counts (*y*-axis) are normalized by the coverage. Mean read counts are used for $n = 10$ patients, whose biological replicates are available. Patient P7, who exhibit the highest expression, is shown separately.

B RT–PCR products, spanning exons 10–15 of *DAB1*, run on the 1.3% agarose gel. *DAB1*(+) patients P7 and P9, and T-ALL cell lines CCRF-CEM, JURKAT and MOLT3 show the expression of unannotated exon. Three bulk thymi, *DAB1*(−) patients P4 and P27, and cell line DND41, which had as low expression as the *DAB1*(−) patients as shown by qPCR, do not show the expression.

 © 2020 The Authors
